# Systematic permutation testing in GWAS pathway analyses: identification of genetic networks in dilated cardiomyopathy and ulcerative colitis

**DOI:** 10.1186/1471-2164-15-622

**Published:** 2014-07-22

**Authors:** Christina Backes, Frank Rühle, Monika Stoll, Jan Haas, Karen Frese, Andre Franke, Wolfgang Lieb, H-Erich Wichmann, Tanja Weis, Wanda Kloos, Hans-Peter Lenhof, Eckart Meese, Hugo Katus, Benjamin Meder, Andreas Keller

**Affiliations:** 1 Chair for Clinical Bioinformatics, Saarland University, Saarbrücken, Germany; 2 Department of Genetic Epidemiology, University Münster, Münster, Germany; 3 Department of Internal Medicine III, University Hospital Heidelberg, Heidelberg, Germany; 4 Institute of Clinical Molecular Biology, Christian-Albrechts-University of Kiel, Kiel, Germany; 5 German Center for Cardiovascular Research (DZHK), Heidelberg, Germany; 6 Institute of Epidemiology and Biobank popgen, Christian-Albrechts-University Kiel, Kiel, Germany; 7 Helmholtz Center Munich, Institute of Epidemiology I, Munich, Germany; 8 Institute of Medical Informatics, Biometry and Epidemiology, Chair of Epidemiology, Ludwig Maximilians University, Munich, Germany; 9 Chair for Bioinformatics, Saarland University, Saarbrücken, Germany; 10 Department of Human Genetics, Saarland University, Saarbrücken, Germany; 11 Klaus Tschira Institute for Integrative Computational Cardiology, Heidelberg, Germany

**Keywords:** DCM, UC, GWAS, Permutation tests, Pathway analysis

## Abstract

**Background:**

Genome wide association studies (GWAS) are applied to identify genetic loci, which are associated with complex traits and human diseases. Analogous to the evolution of gene expression analyses, pathway analyses have emerged as important tools to uncover functional networks of genome-wide association data. Usually, pathway analyses combine statistical methods with *a priori* available biological knowledge. To determine significance thresholds for associated pathways, correction for multiple testing and over-representation permutation testing is applied.

**Results:**

We systematically investigated the impact of three different permutation test approaches for over-representation analysis to detect false positive pathway candidates and evaluate them on genome-wide association data of Dilated Cardiomyopathy (DCM) and Ulcerative Colitis (UC). Our results provide evidence that the gold standard - permuting the case–control status – effectively improves specificity of GWAS pathway analysis. Although permutation of SNPs does not maintain linkage disequilibrium (LD), these permutations represent an alternative for GWAS data when case–control permutations are not possible. Gene permutations, however, did not add significantly to the specificity. Finally, we provide estimates on the required number of permutations for the investigated approaches.

**Conclusions:**

To discover potential false positive functional pathway candidates and to support the results from standard statistical tests such as the Hypergeometric test, permutation tests of case control data should be carried out. The most reasonable alternative was case–control permutation, if this is not possible, SNP permutations may be carried out. Our study also demonstrates that significance values converge rapidly with an increasing number of permutations. By applying the described statistical framework we were able to discover *axon guidance*, *focal adhesion* and *calcium signaling* as important DCM-related pathways and *Intestinal immune network for IgA production* as most significant UC pathway.

## Background

Genome wide association studies (GWAS) examine a substantial set of common genetic variants in larger cohorts of individuals in order to associate single variants or sets of variants with biological traits. Hence, GWAS are usually able to detect significant associations between single nucleotide polymorphisms (SNPs) and human diseases. Since the publication of the first GWAS less than one decade ago in 2005 (e.g. [1] and [2]), far over 1,000 GWAS have been carried out and published. The “Catalog of Published Genome-Wide Association Studies” [3] covers only those GWAS attempting to assay at least 100,000 SNPs in the initial stage and furthermore considers only SNP-trait associations with p-values < 1.0 × 10^-5^. This catalogue lists currently (May, 13th, 2014) 1,920 different papers in PubMed for 1,079 different traits/diseases with 13,380 associations between variants and the respective traits (for each publication at most 50 SNPs are considered). Among the most comprehensive GWAS considering the screened sample size, Teslovich and co-workers [4] investigated the genome for common variants associated with plasma lipids in more than 100,000 individuals of European ancestry and reported over 95 significantly associated loci.

The evolution of GWAS analysis can be compared to the past evolution of expression microarray analysis. While in first instance the expression of restricted sets of genes has been analyzed, more substantial sets of gene expression have been investigated later, and finally, more sophisticated bioinformatics approaches have been implemented to understand the biological importance and relevance of the high-throughput gene expression data. To this end, a large set of gene set enrichment tools and pathway analysis programs was developed such that pathway analyses are now a standard for gene expression studies (no matter whether expression data are generated through microarrays or high-throughput transcriptome sequencing). Historically, over-representation analysis (ORA) was the first method applied, which statistically evaluates the fraction of genes (e.g. all significantly over-expressed genes in a certain disease entity) in a particular biochemical pathway and compares it to a background distribution (e.g. all screened genes in the study that are on the same pathway). Then for each separate pathway a significance value is calculated based on common test statistics, e.g., Hypergeometric distribution, binomial distribution or chi-square distribution. Holmans and co-workers have published a similar example of a respective method, however not relying on a standard distribution [5]. In their ALIGATOR approach significant SNPs are mapped to significantly associated genes, each gene is however counted only once regardless of the total number of significantly associated SNPs. To calculate significance values SNPs were drawn randomly from all SNPs such that the genes containing this SNP were added to the list of significant genes. Overall, 5,000 random gene lists were generated and empirical p-values were calculated for all GO categories with more than 2 significant genes. Following these over-representation methods, Functional Class Scoring (FCS) approaches were developed by the scientific community [6], where first a gene-level statistic is calculated (e.g. relying on ANOVA, Q-statistic, signal-to-noise ratio, *t*-test, WMW-test or Z-scores). Next, the gene-level statistics is aggregated into a pathway level statistics. Here, one of the most commonly applied approaches is the Gene Set Enrichment Analysis [7] (GSEA), which relies on a Kolmogorov-Smirnov-like test statistic. To determine the significance level either a self-contained null hypothesis can be applied where class labels are permuted, or a competitive null hypothesis can be applied where gene labels for each pathway are permuted, and the set of genes in the pathway is compared to a set of genes that are not in the pathway. While usually significance scores have to be calculated by permutation tests, at least in the case of an unweighted gene set enrichment analysis, an exact calculation using dynamic programming has been developed [8]. GSEA approaches that originally were applied in gene expression studies have already been successfully adapted to GWAS [9]. To carry out over-representation analysis (ORA) and FCS approaches a manifold of different stand-alone as well as online tools has been developed over the past decades. A review by Huang and co-workers lists as much as 68 different computational tools that were developed until 2008 [10].

Notably, ORA as well as FCS in their basic implementations do not consider pathway topologies but only sets of genes. Here, genes that are on different parts of the network have the same meaning as genes that are directly influencing each other. Since the direct relation and interaction of genes can potentially add value to the gene set analysis, a third generation of bioinformatics tools has been implemented, covering the topology of pathways. One class of tools combined classical algorithms such as GSEA with pathway topology as implemented in the FIDEPA algorithm [11]. Other examples of pathway topology based algorithms include impact factor based methods [12], NetGSA [13], ScorePAGE [14]. Recently, we published an integer linear programming approach for detecting significantly dysregulated pathways in gene expression data [15,16].

While gene set and pathway analyses have become a standard for gene expression profiling, only a fraction of published GWAS studies made use of such analyses. There is a particular challenge as described by Khatri *et al.*[6], namely low resolution biological resources. While GWAS data comprises the different genotypes for each SNP, the majority of knowledge bases (such as KEGG [17], MetaCyc [18] or Reactome [19]) specify which genes are actively involved in a particular pathway. Thus, first a SNP to gene and then a gene to pathway mapping has to be carried out. To this end, several approaches exist, for example, in the case of the “Pathways of Distinction Analysis” (PoDA, [20]) just the most significant SNP is considered for each gene in order to get a single reference per gene. This however means that the respective SNP is *not* necessarily significantly associated with the considered disease. Besides comprehensive scoring approaches such as SPOT [21], another straightforward approach treats genes as significant where at least one significant SNP has been detected. A comprehensive comparison of several algorithms for pathway analysis using Crohn’s Disease is presented in [22]. Liu *et al.* evaluate ORA and GSEA approaches for Alzheimer Disease [23]. Additional approaches are listed in the review by Wang *et al.*[24].

Additionally, although cohort sizes of GWAS studies are very large and frequently thousands of patients are screened, no SNP may pass genome wide significance after adjustment. This may be due to the fact that the considered trait actually does not depend on genetics in the respective study or that the effect sizes are too small. Here, pathway analysis can contribute to improve the power, while the single genes are not significant the overall pathway might be significant.

For all approaches it is essential to identify real associations and reject as many false positive results as possible. In the present study, we systematically explore the effect of different permutation tests in two sets of GWAS data. The most common approach is permuting the case–control status (column permutations). However, frequently raw data are not available but rather aggregated SNP information. In addition, for web-based applications, uploading of raw data that are required for permuting case–control status can be too time-consuming. Thus, we also evaluated strategies that do not require the case–control status, including permuting significance values of the original case–control status (row permutation I) and randomly permuting the gene labels instead of the significance values of SNPs (row permutation II). In the latter case, the LD is maintained and the sizes of random gene sets correspond to the original size of gene sets. Beyond testing the different permutation test strategies, we assess the required number of permutations to reach statistically stable results. The pathway computations were conducted exemplary on two GWAS datasets for Dilated Cardiomyopathy (DCM) and for Ulcerative Colitis (UC) using the public gene set analysis toolkit GeneTrail [25,26]. Our study addresses the questions, which permutation strategy should be applied to GWAS data and how many permutations are required in order to reach reliable results. In addition, our combined analysis strategy provides novel insights into the molecular pathways involved in DCM.

## Results

### Influence of permutation tests on the number of significant genes

**Figure 1:**
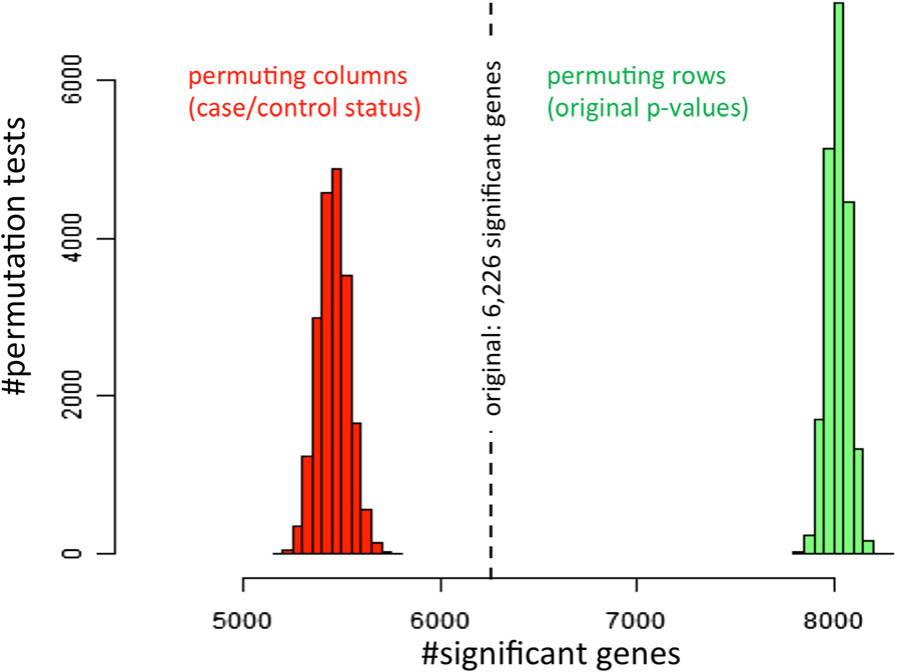
**The two distributions represent the result of the column and row I permutation test approach.** The original data set revealed a total of 6,226 significantly associated genes (dashed line). Following permutations of the case–control status (red), a significantly decreased number of genes is discovered to be significant. Following the SNP permutations (row permutations I), a significantly increased number of genes was discovered to be significant. The second row based permutation strategy preserved the number of genes (6,226). The respective gene sets have been used as input for the pathway analysis.

First, we evaluated how different permutation tests influence the number of significant genes. As lead application we employed our method to a GWAS dataset of 909 patients suffering from Dilated Cardiomyopathies (DCM) and 2,120 population-based controls. As first analytical step, we matched all SNPs to the respective genes according to the information provided by the manufacturer. When one SNP mapped to multiple genes, all genes were taken into account. Next, genes were considered as significant, if at least a single SNP was discovered in that gene (significance value of p < 0.05, adjusted for GC and covariates). The SNPs were not adjusted for multiple testing since a standard Bonferroni correction did not yield any individual genome-wide significant SNPs in this study. For the original data set we calculated 6,226 significantly associated genes. By carrying out 20,000 permutation runs across the columns of the GWAS matrix, corresponding to permutation of case–control status, we found a significantly decreased (z-score based p-value <10^-4^) number of genes in the range of 5,500 genes per permutation test, as indicated by the red distribution in the Histogram plot (Figure 1). We likewise carried out 20,000 permutations across the rows of the GWAS matrix, corresponding to randomly permuting the significance values per SNP. Hereby we calculated a significantly increased (z-score based p-value of <10^-4^) number of significantly associated genes (around 8,000 per permutation test run), as demonstrated by the green distribution in Figure 1. Altogether, both distributions were significantly different from each other (two-tailed unpaired t-test of <10^-10^). In the third permutation test strategy, i.e. permuting the genes, the number of significant genes was preserved. The substantial difference between the three analyses is well explained by the completely different permutation approaches. While e.g. for the column permutations correlations between SNPs are obtained, this information is completely lost in the case of permuting SNPs. This fact is of particular importance when hypotheses are tested that combine information across SNPs.

Moreover, we also evaluated the influence of the alpha level on the number of significant SNPs and decreased the threshold to 0.01, 0.001, 0.0001 and 0.0001, respectively. In this analysis, we found a rapidly decreasing number of significant genes although we define a gene as significant if it contains just a single significant SNP. Specifically, the number of genes decreased to 39.7%, 7.5%, 1.3% and 0.2% with just 13 genes remaining for the lowest threshold of 0.0001.

### Influence of permutation tests on pathway analysis

Next, we explored the influence of the different permutation strategies for GWAS pathway analyses relying on the Hypergeometric distribution. By using GeneTrail, we investigated 241 different biochemical pathways from the KEGG database and studied whether more or less genes than expected by chance are located on each pathway. The respective pathways are then denominated as enriched or depleted, respectively. While the depleted pathways contain the genes that are not affected by the disease, the enriched pathways are significantly altered. Therefore, here we focus on enriched pathways and provide the depleted pathways for completeness.

Analogously to the single gene analysis, we evaluated the influence of the alpha level on the pathway analysis to calculate significant SNPs by decreasing the threshold from 0.05 to 0.01, 0.001, 0.0001 and 0.0001, respectively. Please note that only the significance level for identification of SNPs has been varied, while the threshold to discover significant pathways was in all analyses 0.05 following adjustment for multiple testing. For the original alpha level of 0.05 (gene set size: 6,226), we calculated 54 significant pathways after adjusting for multiple testing. By considering SNPs with significance below 0.01 (gene set size: 2,470), just 11 enriched pathways remained. When increasing the stringency of the threshold to 0.001 (gene set size: 466), no significant pathway remained. These results suggest that 0.05 or 0.01 are reasonable thresholds. The results in the manuscript are based on the least stringent alpha level of 0.05.

For each pathway, we calculated four different significance values. Two significance values correspond to the two distributions described above and outlined in Figure 1 (column permutations, row permutations I). The third p-value corresponds to the permutation of genes (row permutation II) and the fourth p-value corresponds to the original data, respectively. In the latter case, significance values were computed using the Hypergeometric distribution and significance values were adjusted for multiple testing using the Benjamini Hochberg approach [27]. For the permutation tests, we calculated a significance score for each pathway *p* as the fraction of all 20,000 column and row permutation tests with higher significance for pathway *p* as the original data set. The significance values resulting from the four sets of pathway analysis are presented as bar chart in Additional file 1: Figure S1. While column permutation tests (average p-value of 0.33) and row permutation tests I (average p-value of 0.36) were clearly less significant than the original results (average p-value of 0.24), the second row permutation test strategy showed substantially smaller p-values (average p-value of 0.07). As two-tailed paired t-tests indicate, the difference between original p-values and row permutations I was higher (2*10^-13^) than the dissimilarity between original p-values and column permutations (2*10^-10^). The highest difference was however calculated for row permutations II with a p-value of < 10^-16^. Although row I and column permutation tests showed a slightly higher concordance to each other, the difference between both approaches was still significant (5*10^-7^). All significance values for all pathways and all permutation tests are provided in Additional file 2: Table S1.

The original motivation for permutation tests is to cross-check the p-values obtained by classical tests such as the Hypergeometric distribution to discover putative false positive pathways. Based on the results above, we conclude that column permutation as well as row I permutation tests highlight relevant pathways. In contrast, row permutation tests II in all cases confirmed the results of the Hypergeometric test, even with substantially lower significance values, not adding to the specificity of the pathway analysis.

**Figure 2:**
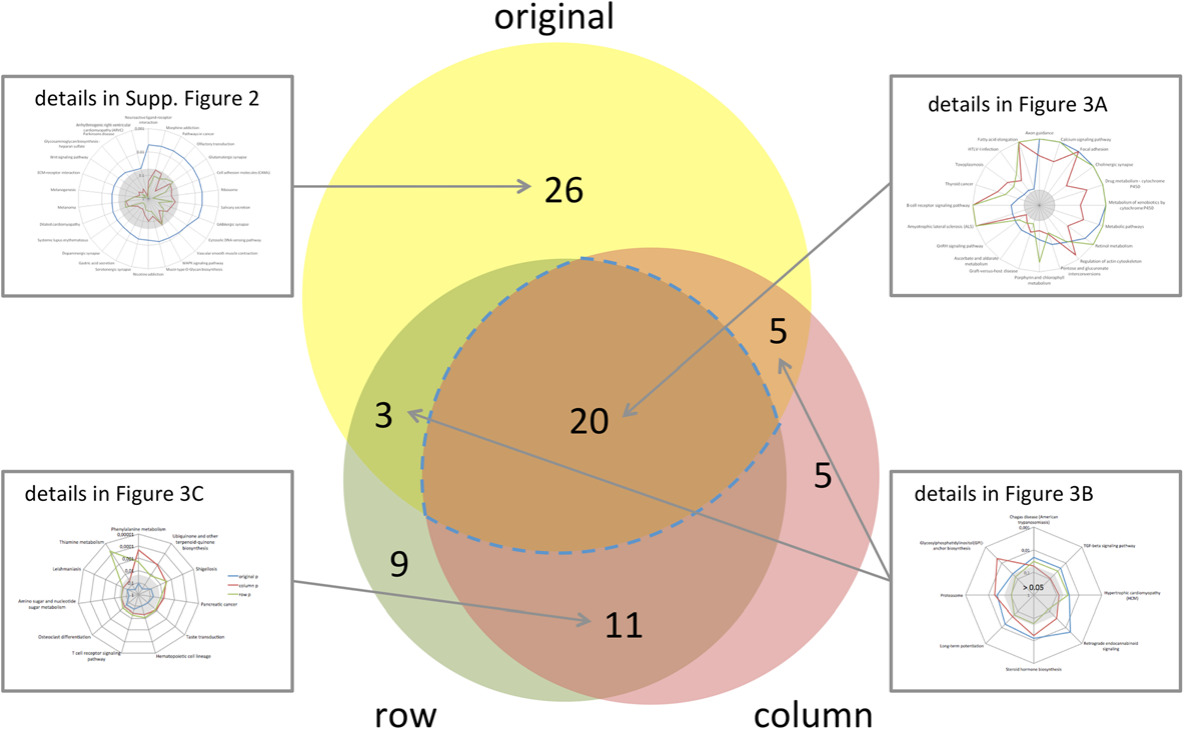
Venn diagram showing the overlap between the three different approaches.

**Figure 3:**
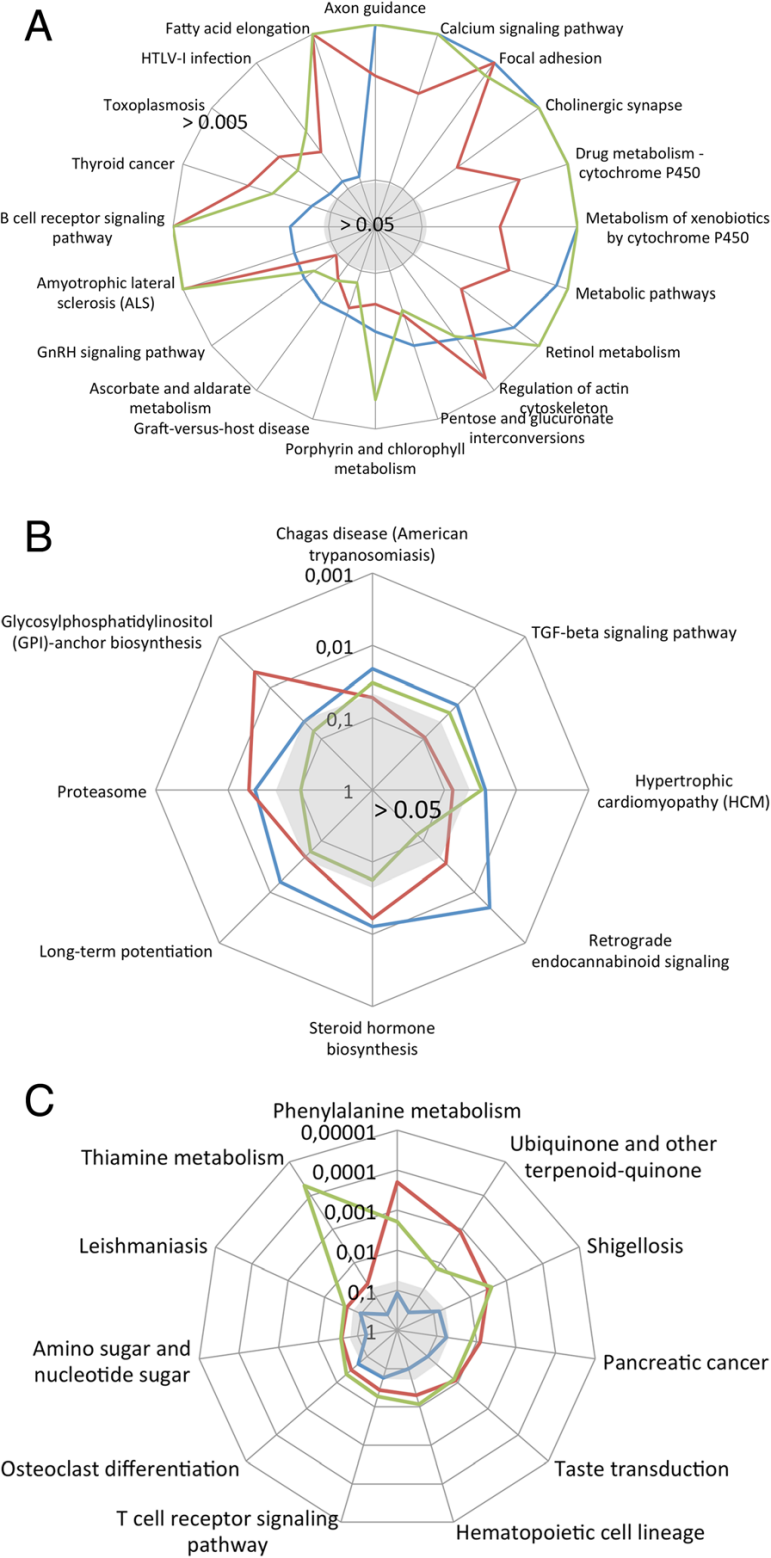
**Overview on the 20 significant pathways across all approaches (Figure 3A), in both permutation tests (Figure 3B) and just in original calculations (Figure 3C).** The figure presents the significance values for the 20 pathways (ordered clockwise according to decreasing significance as calculated by the Hypergeometric test), showing p-values < 0.05 for all three approaches. The further away from the middle the higher the significance scores (on a logarithmic scale). The grey shaded area in the middle corresponds to non-significant pathways. Significance values have been cut at 10^-5^.

Consequently, we focus in the following on the interpretation of column permutation tests and row permutation tests I. To understand the differences between the Hypergeometric test and the two remaining permutation tests, we calculated the overlap in significant pathways. As presented in the area proportional Venn diagram in Figure 2, 79 distinct KEGG pathways were significant in at least one of the three tests. The highest number of significant pathways was discovered for the original data (54), while permutation testing for columns and rows revealed 41 and 45 significant pathways, respectively. Remarkably, the overlap between all three tests was substantial, with 20 pathways remaining significant in all three tested scenarios. The most significant of these pathways included “axon guidance”, “calcium signaling” and “focal adhesion”. All 20 pathways that remained significant in the three analyses are shown in Figure 3. Here, the distance from the center reflects each pathway’s significance, where larger distances correspond to increased significance. All pathways outside of the area represented in the center of Figure 3 are significant at a threshold of p = 0.05. As this figure shows, the concordance between permutation of rows and columns appears generally high, at least for the subset of 20 pathways, as demonstrated by a correlation of 0.84. As mentioned above, some pathways were highly significant (p-value <0.005). Notably, the three most significant pathways with respect to the original distribution belonged to the 20 pathways being significant in all three tests such that row- as well as column permutations confirmed the original results. These networks contain “axon guidance”, “calcium signaling pathway” and “focal adhesion” with adjusted p-values of below 10^-5^ (original set). As detailed in the discussion section, all three pathways are important key networks for cardiovascular disorders. Besides these, further 26 pathways have been excluded by both approaches, being significant just in the original data set results (Additional file 3: Figure S2).

Again, column and row permutation tests I showed a generally good concordance in marking potentially interesting candidate pathways such as “vascular smooth muscle contraction” or “dilated cardiomyopathy” as likely false positives. Notably, for these pathways the second row permutation test strategy found the pathways as highly significant. In case of “dilated cardiomyopathy” the original p-value was 0.02 while row permutations II reveal a p-value of 0.0003. For “vascular smooth muscle contraction” the original p-value was 0.01 and for row permutations II as low as 10^-4^. A potential reason is a size bias since the genes included in both networks are substantially longer compared to the average length of human genes (p-values according to Wilcoxon Mann–Whitney test of 2*10^-8^ and 2*10^-6^, respectively), demonstrating that the applied column and row I based permutation approaches effectively handles this size bias while the second row based permutation strategy does not.

**Figure 4:**
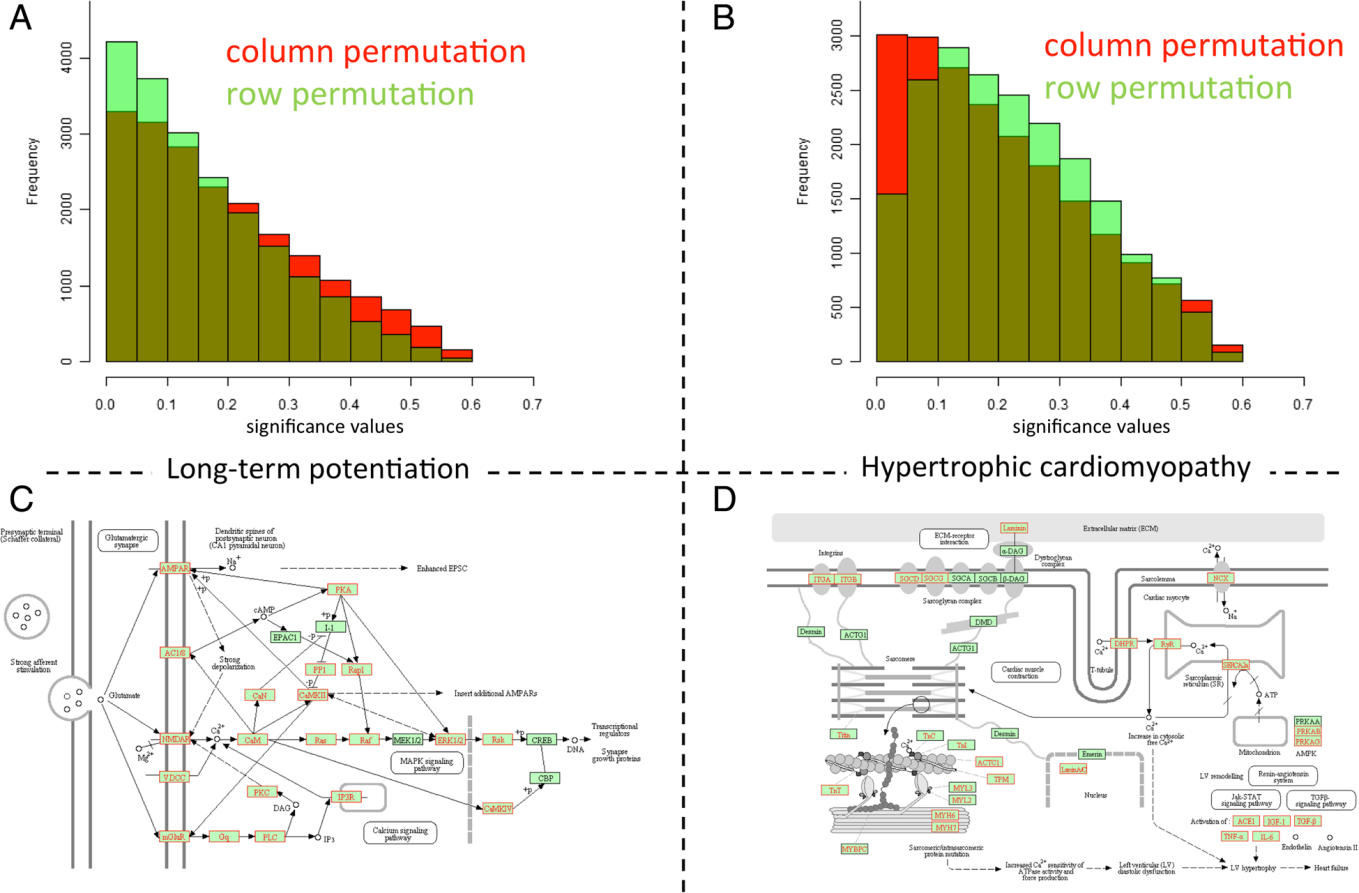
**Difference between row- and column permutations.** The histograms in panel **A** and **B** show for two pathways the significance values as calculated for row and column permutations, respectively. Panels **C** and **D** present the respective pathways as provided by KEGG. Here, red marked genes correspond to significant genes in our GWAS.

Our analyses suggest that row I and column permutations provide fully concordant results and that one of the two approaches will be sufficient. Nevertheless, while for the total set of networks included in the Venn diagram in Figure 2 (all pathways that are significant at least in a single test) in 31 cases row and column permutation tests were concordant according to an alpha level of 0.05 and further 26 pathways were rejected by both strategies, in as much as 22 cases discordance between row I and column permutation tests was observed. Specifically, in 5 cases only the original analysis and column permutations were significant. In 3 cases only the original analysis and row permutations were significant (details are provided in Figure 3B); in 5 and 9 cases, only row permutations or respective column permutations were significant. Notably, in as many as 11 cases significant results in row and column permutations were discovered while original results did not show any significance (see Figure 3C). It is however worth mentioning that a substantial fraction of these 11 paths still exhibited low p-values, e.g. “Osteoclast differentiation” and the “T cell receptor signaling pathway” slightly missing the alpha level in the original analysis with p-values of 0.051 and 0.057. A total of 7 pathways revealed Hypergeometric test p-values below 0.1 (see Additional file 2: Table S1).Since row I and column permutation results do not agree in all cases, a detailed consideration of the results is required. An example where row permutation tests yielded a significant enrichment while column permutations revealed a higher and non-significant value includes the “long term potentiation”, as presented in Figure 4 (panels A and C). Vice versa, panels B and D of that figure visualize an example where column permutations provided a significant result while row permutations were not significant (hypertrophic cardiomyopathy, HCM). In both cases, large parts of genes participating in the pathways are significant in the GWAS, highlighted in red in the representations on the lower part of Figure 4. Additional 11 pathways where the Hypergeometric tests did not yield any significant result, but permutation tests did, include Thiamine, Phenylalanine metabolism, Shigellosis, Hematopoietic cell lineage, Taste transduction, Pancreatic cancer, Ubiquinone and other terpenoid-quinone biosynthesis, T cell receptor signaling, Osteoclast differentiation, Leishmaniasis and Amino sugar and nucleotide sugar metabolism. These pathways are however only loosely connected to DCM.

**Figure 5:**
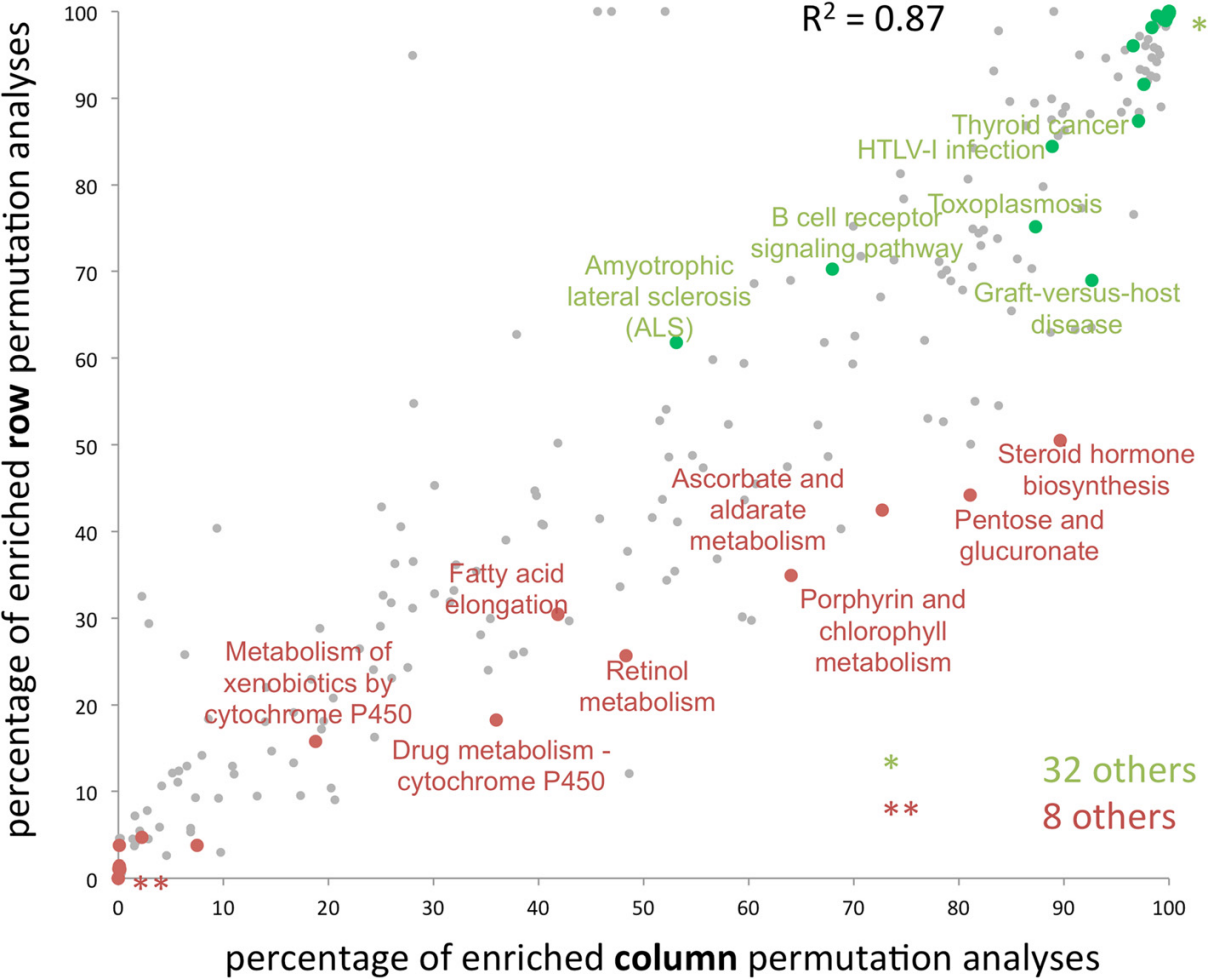
**Comparison between enriched and depleted pathways.** Each dot corresponds to one pathway. Red dots correspond to depleted and green dots to enriched pathways.

Our analyses considered significantly enriched as well as depleted pathways, representing both tails of the distribution of all permutation tests. In many cases it makes sense to treat enriched and depleted pathways separately from each other, corresponding to a one-tailed analysis. While the enriched pathways are most affected by the disease, depleted pathways may provide information on molecular networks that are not affected by the trait of interest. We thus calculated for each of the 241 pathways how many percent of the row and column permutation tests are enriched and depleted. As shown in Figure 5, row and column permutation tests revealed on average a good correlation as indicated by the R^2^ value of 0.87. Here, the significantly enriched and depleted pathways are highlighted in green and red. Notably, many pathways are enriched or depleted in almost all permutation test runs including 32 pathways that are 100% enriched and located in the upper right corner in Figure 5, and 8 pathways that are 100% depleted and located in the lower left corner of this figure. The pathways in the upper right corner also contain the three previously described pathways “axon guidance”, “calcium signaling pathway” and “focal adhesion”. Remarkably, four pathways are clear outliers in Figure 5 (upper middle part of the diagram), containing “Cyanoamino acid metabolism”, “fatty acid biosynthesis”, “vitamin B6 metabolism” and “butirosin and neomycin biosynthesis”. These are likely false positives due to a limited number of SNPs in the genes of the respective pathways or a small number of participants in the pathway. To effectively adjust for such artifacts, the analysis could be restricted on larger pathways, however, leading to a loss of information on smaller paths. All significance values for enriched and depleted pathways are provided in Additional file 4: Table S3.

### Required number of permutation tests

**Figure 6:**
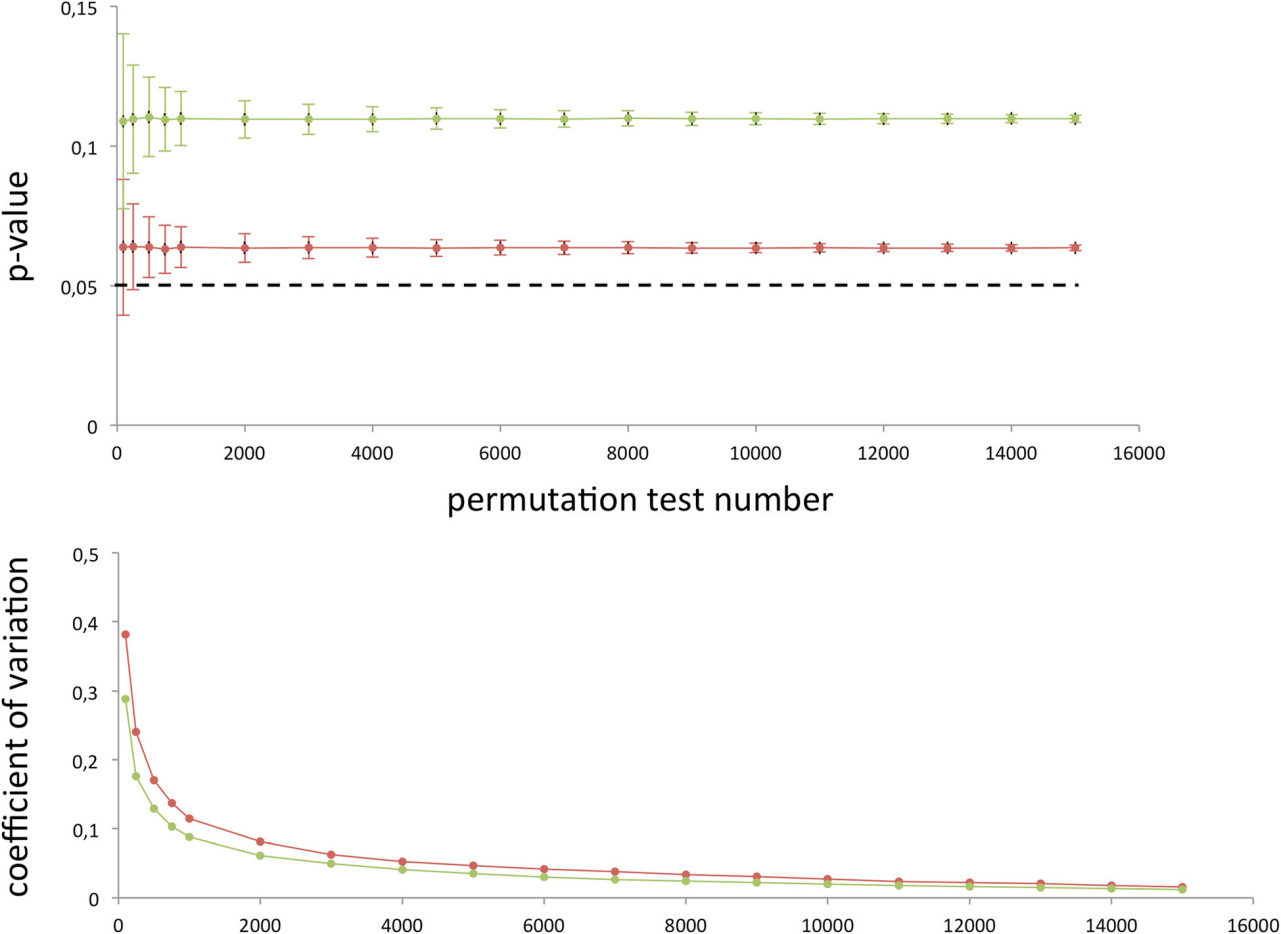
**Influence of the number of permutations.** The upper panel of the figure shows for column (red) and row (green) permutation tests the average significance value and the standard deviation for “Pathways in cancer”. The lower panel shows the coefficient of variation (CV) for both approaches.

Another important question in GWAS pathway analysis is how many permutations have to be carried out in order to obtain stable results with respect to the considered pathways? Here, one common choice is to generate 1,000 different permutations, just a small fraction of the exponentially growing permutation number. We explored the Coefficient of Variation (CV), the ratio of the standard deviation to the mean as potential criterion for estimating the required number of permutations. In detail, we started by sampling 100 of the 20,000 permutation tests and stepwise increased the number. For each permutation set size 1,000 random drawings were carried out to calculate average value, standard deviation and CV value for column as well as row permutations. First, we considered the average and standard deviation for all pathways with 1,000, 2,000 and 5,000 permutation tests for row and column permutations separately. Additional file 5: Figure S3 shows exemplarily the dependency between column permutation test number and CV. Particularly for the significant pathways on the left of the vertical black line (p = 0.05), the difference between 2,000 permutations (blue) and 5,000 permutations (green) was not significantly larger than between 1,000 and 2,000 permutations. To exactly assess at which number of permutations the significance values converge for a certain pathway, we estimated the influence of the number of column and row permutations on the significance for “pathways in cancer”. Figure 6 presents the average significance score and the respective standard deviation for up to 15,000 of these permutations in the upper panel. In the lower panel of that figure the coefficient of variation for both, column and row permutations, is presented. Here, it can be seen that significance values converge rapidly, resulting in our example in a moderate coefficient of variation, such that in our case indeed 2,000 permutations were sufficient to estimate the actual significance value in a reasonably small confidence interval and coefficients of variation of approximately 0.1.

### Ulcerative Colitis (UC) pathways

To validate our approach, we evaluated GWAS data measured from Ulcerative Colitis (UC) patients. Analogous to our results in the previous chapters, we focused on the most complex permutation test approach and carried out 10,000 permutations of the case–control status permutations. Following our original analysis strategy, we selected an alpha level of 0.05 to consider a SNP as significant. For the original data set we calculated 7,082 significant genes. In line with the results for DCM, we also found a significantly decreased number of genes in the permutation tests with an average of 6,775 genes.

In the enrichment analysis we discovered as much as 51 KEGG pathways to be significant following adjustment for multiple testing at an alpha level of 0.05. In the subsequent evaluation of the permutation tests, 30 of these pathways (59%) were marked as potentially false positive paths and 21 remained significant. The two most significant networks with p-values of 6*10^-4^ and 8*10^-4^, respectively were “Intestinal immune network for IgA production” and “Toxoplasmosis”. The next pathways with significance values of 0.001, 0.002 and 0.003 contain “Maturity onset diabetes of the young”, “Fat digestion and absorption” and “Glycerophospholipid metabolism”. The first pathway that has been excluded by the permutation tests was “Cell adhesion molecules”. All significance values are provided in Additional file 6: Table S2.

Next, we evaluated the required number of permutations for the UC data set with the same approach as for DCM. Corresponding to our previous results on DCM, we again did not discover substantial differences for the relevant pathways (p = 0.05). The difference between 2,000 permutations (blue) and 5,000 permutations (green) was not significantly larger than between 1,000 and 2,000 permutations (Additional file 7: Figure S4). Since this analysis revealed that for very significant pathways very small permutation test numbers suffice, we again picked a pathway with a p-value in the range of 0.05 in order to estimate the finally required number of permutations. As an example, we investigated the pathway “RNA polymerase”. As was observed in the case of DCM, the significance values rapidly converged for this pathway. Additional file 8: Figure S5 demonstrates that, again, significance values rapidly converge with increasing permutation test number and that for permutation test numbers between 1,000 and 2,000 coefficients of variation below 10% can be obtained. Generally, the results calculated for UC matched well to the results obtained for DCM.

### Comparison of DCM and UC

Finally, we investigated on the overlap of DCM and UC genes and pathways. Of the 7,082 genes calculated for UC, 3,919 (55%) were likewise detected for DCM, representing a substantial overlap. Considering the pathways that are significant according to the Hypergeometric distribution, still 24 of the 51 UC pathways are overlapping with DCM (47%). After applying the permutation tests, however just 2 of the 21 pathways are overlapping between both diseases (10%). The respective pathways are “GnRH signaling pathway” as well as “Toxoplasmosis”. This analysis indicates that the permutation tests filter out a substantial part of false positive pathway candidates.

## Discussion

Pathway analysis for GWAS has already been applied to various diseases such as pancreatic cancer [28], type 2 diabetes [29], Alzheimer [23], non-syndromic cleft lip [30] and many others. In our study we explored pathways in a GWAS of dilated cardiomyopathy and at the same time systematically evaluated different permutation test strategies. While we obtain reliable results using a gene-set based approach, relying on an over-representation statistics which is calculated via the Hypergeometric test, topology based methods such as scorePAGE [14] or optimization based algorithms [15,16] should be considered to improve the signal to noise ratio in GWAS and enhance the systems understanding of human pathogenic processes. Yet, there remain several challenges in GWAS pathway analysis:

The first challenge is that existing pathway resources such as KEGG having a lower resolution and comprising relatively few genes compared to genome-wide SNP datasets. Additionally, GWAS consider multiple variants in each single gene and even more variants in non-coding regions. Thus, associated variants first have to be assigned to genes and significance scores per gene have to be calculated. Here, various algorithms such as SPOT [21] have been developed. Straightforward approaches treat genes as significant where at least *x* significant SNPs have been detected in that gene. This however may introduce a bias towards longer genes, as we demonstrate in our study. Here, the permutation tests of rows and columns were very effective to account for potential size bias. Other approaches that could be applied in order to take gene size into account are to normalize the number of significant variants per gene by the gene length or by the total number of variants on this gene included in the study. We explored the respective approaches but did not discover improved results compared to the straightforward method employed in our study.

Another challenge is significance value calculation of permutation tests and permutation test numbers to be carried out. Generally, the significance score for permutation tests is calculated as fraction of all permutations with more significant result than in the original analysis. To avoid p-values of zero, the minimal significance score to be reached by this method is 1/(# of permutations). One approach to account for this is to calculate p-values based on tail approximation. Knijnenburg and co-workers [31] present an algorithm where the tail of the distribution of permutation values is approximated by a generalized Pareto distribution, which accurately estimated significance values. Reducing the number of permutations is of special importance when considering many different biological categories. For our approach few thousand permutations were sufficient in order to gain valuable insights into the molecular pathogenesis of DCM. For our ORA based approach it was however essential to permute the case–control status as well as the significance values of single SNPs. A similar claim has already been made by Efron and Thibsirani [32] who address the problem of identifying differentially expressed groups of genes from a microarray experiment based on the gene set enrichment analysis (GSEA).

Despite these challenges, pathway analysis helps to understand pathogenic processes on a molecular level. By applying the ORA based pathway analysis for DCM we detected association signals to be enriched in different pathways indicating their modulation by common variants. Most importantly, three very highly significant pathways (adjusted original p-values below 10^-5^) that remained significant after column and row permutation tests were discovered, including “axon guidance”, “calcium signaling pathway” and “focal adhesion”. The “focal adhesion pathway” for instance is an interacting network of proteins that is essential for maintaining cardiomyocyte integrity [33], mechanosensing, and mechanotransduction [34-36]. Perturbations in this pathway have been observed following chronic alterations in cardiac afterload and maladaptive remodeling [37], all important in the pathogenesis of DCM. While calcium signaling is very obvious to be important for DCM - a disease with the hallmark of disturbed calcium homeostasis - axon guidance, which was most substantially enriched, represents a more surprising finding. It may indicate a possible link between DCM and abnormalities in cardiac innervation. For instance, chronic heart failure and its progression are associated with increased sympathetic tone, decreased vagal control, and regional variability in innervation [38,39]. The components of the axon guidance pathway are also involved in cardiac development and differentiation [40,41]. Moreover, the maintenance of a normal cardiac function depends on the autonomic nervous system, characterized by an intricate balance between the sympathetic and parasympathetic activity. Not only do they regulate the cardiac conduction system, but also orchestrate heart rate and force of contraction. In congenital heart diseases as well as cardiac ischemia and heart failure, we can find altered cardiac innervation, with their underlying developmental and regulatory mechanisms. Vascular sympathetic innervation is an important determinant of blood pressure and blood flow, with recent data suggesting that vascular endothelial cells (EC) express semaphorin 3A (SEMA3A), a repulsive axon guidance cue. As such, Damon et al. have looked closely at rat aortic vascular ECs expressing SEMA3A as well as other class 3 semaphorins and found out that vascular EC-derived SEMA3S inhibited sympathetic axon growth [42]. Moreover, Fish et al. looked at the interaction of members of the Slit family of secreted ligands with Roundabout (Robo) receptors, which provide guidance cues for many cell types. The Slit-Robo signaling pathway is involved in the development of the pericardium, the sinus horn myocardium, and the alignment of the caval veins. In zebrafish, miR-218 and multiple Slit/Robo signaling components are required for heart tube formation [40]. Mommersteeg et al. uncovered that reduced Slit3 binding in the absence of Robo1 led to an impaired cardiac neural crest survival, adhesion, and migration, with pericardial defects created by abnormal localization of the caval veins combined with ectopic pericardial cavity formation [43]. In diseased hearts, nonuniform innervation promotes enhanced sympathetic activity and therefore life-threatening arrhythmias. Miwa and collaborators demonstrated that GDNF promotes sympathetic innervation in both native cardiac cells as well as stem cell-derived cardiac cells, with enhanced abnormal sympathetic innervation in pathological conditions such as myocardial infarction or heart transplantation due to sympathetic “nerve sprouting” as well as disordered reinnervation [44].

We also need to carefully consider the overlap of different pathways since biochemical networks e.g. from the KEGG database are usually not disjoint. While no gene was located in all three pathways we detected 4 genes common between “axon guidance” and “calcium signaling” (PPP3R1, CHP2, PPP3CA, PPP3CC), 6 genes in common between “calcium signaling” and “focal adhesion” (PRKCB, PDGFRA, PRKCA, MYLK4, MYLK, EGFR) as well as 11 genes in common between “axon guidance” and “focal adhesion” (PAK1, GSK3B, PAK6, ITGB1, PAK7, FYN, CDC42, ROCK1, ROCK2, MAPK1, PTK2), representing likely key-players for DCM. After removing overlapping genes respectively and repeating the same analysis, the pathways did not remain significant, demonstrating that these genes are of central importance for the pathogenesis of DCM in these three pathways. To further explore the role of these pathways we removed all genes from the respective networks and repeated the analysis. The results demonstrated a substantial shift with much less significant results. The only category which remained significant in this analysis and was also significant in the original results and all permutation test approaches, was “Graft-versus-host disease”. These results imply that not only the overlapping genes but also all genes on the respective paths and, thus, the pathways themselves play a crucial role in pathogenesis of DCM.

Permutation tests help to filter paths, which are strongly significant in standard analyses such as the Hypergeometric test, improving the specificity of network analyses. Remarkably, already the fourth most significant pathway in our original analysis, “neuroactive ligand-receptor interaction” with as many as 124 genes located within this network and being highly significant (p-value of 9*10^-6^) was ruled out by both permutation test strategies, where 3,564 permutations of case–control status and 7,510 permutations of original association p-values showed higher significance than the originally calculated 9*10^-6^.

It is noteworthy that the permutation test strategies led to significant results in 11 cases while the originally applied ORA analysis did not reveal a significant result. Some of the 11 paths showed still low p-values in the range of 0.05 to 0.1. Nevertheless, few of these 11 pathways are related to heart failure at all. Genes on the T cell receptor signaling pathway for example have shown to categorize heart failure patients into three risk groups [45]. The fact that the majority of these pathways is not related to dilated cardiomyopathies further supports our hypothesis that the consideration of Hypergeometric test along with both permutation test strategies lead to the most reasonable results from a biological perspective.

We repeated the most promising analysis strategy with UC as second disease. After applying column-based permutations we found a set of pathways, which was very different from the DCM networks. Interestingly, we discovered “Intestinal immune network for IgA production” as most significant pathway. Immunoglobulin A (IgA) is an antibody that plays a critical role in mucosal immunity. More IgA is produced in mucosal linings than all other types of antibody combined [46]. In its secretory form, IgA is the main immunoglobulin found in secretions from the gastrointestinal tract. Secretory IgA protects the immunoglobulin from being degraded by proteolytic enzymes, thus IgA can survive in the harsh gastrointestinal tract environment and provide protection against microbes that multiply in body secretions. In the gut, IgA can bind to the mucus layer on top of the epithelial cells to form a barrier capable of neutralizing threats before they reach the cell. Therefore, decreased or absent IgA, termed selective IgA deficiency, is a clinically significant immunodeficiency. Recent genetic studies have shown that a subgroup of patients with mutations in known immunodeficiency genes has severe early onset colitis. Ongoing projects now systematically screen all known Immunodeficiency genes in early onset UC patients for mutations. It is further known that UC patients have a dysregulated gut microbiome, i.e. especially the bacterial diversity is reduced in UC patients. Given the importance of IgA in maintaining intestinal homeostasis [47] and in host-microbe interactions [48], an important role of the IgA pathway in UC disease etiology is likely. All significant genes on the core networks for UC as well as DCM are summarized in Additional file 9: Table S4.

Although our results already revealed interesting biological results, future approaches that integrate the topology of networks rather than sets of genes will enhance the discovery of sub-networks or specific pathways that are significantly perturbed in a certain trait.

It has to be mentioned that the three tested permutation test approaches evaluate different null hypothesis. Particularly, it should be noted that permuting SNPs explicitly does not maintain the LD scattering any single, linked effect among genes and potentially introducing inflation in the null distribution. This effect may become even more important depending on how significant genes are calculated from a list of significant SNPs. Thus, the results of the SNP permutations have to be carefully evaluated and, where possible, case–control permutations should be carried out.

## Conclusions

Our study elucidates that for GWAS permutation of case–control status as well as permutation of the original associations’ p-values are reasonable in order to systematically uncover potential pathogenic pathways for human diseases. Especially in the latter case, results require careful interpretation since this kind of permutation test does not maintain the LD. While the gold standard of permuting case–control status should be carried out, permuting SNPs appears to represent a reasonable alternative when case–control permutations are not possible. The most specific results are obtained for those pathways, where all three approaches yielded significant results. Furthermore we demonstrate that few thousand permutations are sufficient in order to obtain reliable results for our data example. In summary, the following parameters for the GWAS pathway analysis showed reasonable performance in our analysis: significance threshold for SNPs – 0.05; permutation approach – case–control permutations; number of permutations – 2000; significance threshold for pathways – 0.05. Further analyses on other traits will show whether these parameters can be generalized or have to be adapted for other GWAS studies.

## Methods

The GWAS dataset for pathway analyses: data used for pathway analyses was retrieved from Meder et al. [49] Stage 1 (screening phase) of this GWAS on DCM consisted of 909 individuals of European descent with DCM recruited between 2005 to 2008 and 2,120 controls from the PopGen and KORA population-based cohorts. Case–control association tests were conducted assuming an underlying additive genetic model with 1 degree of freedom (df) using the PLINK software package version 1.07 (http://pngu.mgh.harvard.edu/purcell/plink). SNPs exhibiting minor allele frequencies <3%, call rates ≤95%, or deviations from Hardy-Weinberg equilibrium considering a significance level of 0.05 for controls and 0.001 for cases were excluded from further analyses. Analyses were adjusted for sex and age of the included unmatched individuals by means of logistic regression. The genomic inflation factor was calculated as median of all SNPs divided by the median of a chi square distribution with 1 degree of freedom and was used to correct p-values of the association analyses for genomic control (GC) in order to effectively adjust for population stratification [50].

The second GWAS data set on Ulcerative Colitis was extracted from Ellinghaus et al. [51], consisting of 987 UC cases and 2968 healthy controls from the PopGen and KORA cohorts. All probands are of German descent and were genotyped using the Affymetrix Genome-Wide Human SNP Array 6.0 plattform (Affymetrix, Santa Clara, CA). SNPs with a minor allele frequency < 1%, call rates ≤ 95% or significant deviation from HWE in controls (p < 10^-4^) were excluded from further analysis. As for the DCM data set, case–control association tests were conducted using the PLINK software package version 1.07 assuming an underlying additive genetic model with 1 degree of freedom. The analysis was adjusted for Genomic Control by using logistic regression.

Permutation tests: In order to validate the significance of results from pathway analyses, re-sampling approaches are commonly applied. In our study we carried out a permutation of the case–control status (permutation of columns) as well as randomly shuffling the significance value for each SNP (permutation of rows). First, the case–control status has been randomly shuffled 20,000 times and the respective runs have been evaluated according to the methodology described earlier (in the following denoted as column permutations). In order to permute the original associations’ p-values of the GWAS data analysis as described above, original significance values have been randomly assigned to arbitrary SNPs (in the following denoted as row permutations I). The latter procedure ensured that the total number of significant SNPs did not vary between the various permutation test runs. Please note that the number of significant genes nevertheless varies between different permutation test runs. Additionally, we tested a third permutation variant by randomly permuting the gene labels instead of the significance values of SNPs (row permutations II). In this case, the LD is maintained and the sizes of random gene sets correspond to the original size of gene sets.

Remarkably the number of possible permutations between those approaches is substantially different. Considering a GWAS with *x* cases and *y* controls and covering *z* SNPs (or *g* Genes), a total of

$$\left(\begin{array}{c} x+y\\ y \end{array}\right)=\frac{\left(x+y\right)!}{x!y!}$$

different permutations of case–control status are possible while up to *z!* (or *g!*) permutations of SNP significance values (or genes) can be carried out. Notably, for usual GWAS the number of SNPs is considerably higher than the number of screened individuals (*z > > x + y*) such that significantly more row permutations are possible.

In order to calculate a p-value for a pathway *R* based on permutation tests (either row or column permutations) we applied the following approach:

$$p_{\text{perm}}^{R}=\frac{\sum_{n=1}^{N_{\text{tot}}}I\left(p_{n}^{R}\geq p_{\text{original}}^{R}\right)}{N_{\mathrm{tot}}}$$

Here, *p*^*R*^_*n*_ represents the p-value for pathway *R* in the *n-*th permutation test, *p*^*R*^_*original*_ represents the original p-value for that pathway as calculated by the Hypergeometric distribution, *N*_*tot*_ equals the number of permutations carried out (20,000) and *I()* is the indicator function, evaluating to 0 or 1, depending whether the permutation test is less significant as compared to the original p-value. In order to avoid significance values of zero in case that no permutation test is more significant than the original data a pseudo-count can be added.

Pathway analysis: A total of 60,001 different analysis runs have been carried out, three times 20,000 permutation tests for each column and row permutations along with the original data set. All calculations have been carried out with the freely available gene set analysis tool GeneTrail [25]. As biological category 241 different KEGG [17] pathways were considered such that altogether around 10 million analyses were performed. To assess the significance the Hypergeometric test was calculated. Given a total of *g* significant genes of which *k* belong to pathway *R* and a total of *h* genes of which *i* belong to R, the p-value for enriched pathways is calculated as

$$\sum_{j=k}^{g}\frac{\left(\begin{array}{c} i\\ j \end{array}\right)\left(\begin{array}{c} h‒i\\ g‒j \end{array}\right)}{\left(\begin{array}{c} h\\ g \end{array}\right)}$$

and accordingly for depleted pathways as

$$\sum_{j=0}^{k}\frac{\left(\begin{array}{c} i\\ j \end{array}\right)\left(\begin{array}{c} h‒i\\ g‒j \end{array}\right)}{\left(\begin{array}{c} h\\ g \end{array}\right)}$$

After all significance values were calculated, p-values were adjusted for multiple testing using the Benjamini Hochberg approach [27]. All pathways with less than two genes located onto that pathway were excluded from significance value calculation. Besides KEGG pathways, GeneTrail potentially offers to carry out calculation for a substantially larger set of ten thousands of functional biological categories including e.g. Gene Ontology [52], chromosomal position, targets of certain miRNAs, transcription factors from TRANSFAC [53] but also many others.

## Competing interests

The authors declare that they have no competing interests.

## Authors’ contributions

CB carried out permutation tests and pathway analysis, FR did the primary data analysis of GWAS arrays, MS supported the primary data analysis of GWAS arrays, contributed in writing the manuscript, JH and KF participated in the analysis of the GWAS data, AF contributed in writing the manuscript and supported the data interpretation, WL, HEW, TW, and WK participated in the analysis of the GWAS data, HPL contributed in interpreting the data, EM contributed in writing the manuscript and in pathway analysis, HK contributed in study design, BM contributed in study design, data analysis and wrote the manuscript, AK contributed in data analysis and wrote the manuscript. All authors read and approved the final manuscript.

## Supplementary Material

Additional file 1 Overview of the significance values resulting from the four sets of pathway analysis as bar chart. Click here for file

Additional file 2 All significance values for all pathways and all permutation tests for the DCM dataset. Click here for file

Additional file 3 Spider diagram of 26 pathways that have been excluded by both permutation approaches, being significant just in the original data set results. Click here for file

Additional file 4 All significance values for enriched and depleted KEGG pathways. Click here for file

Additional file 5 Comparison of the average and standard deviation for all pathways with 1,000 (black), 2,000 (blue) and 5,000 (green) permutation tests for row and column permutations separately (DCM dataset). Click here for file

Additional file 6 All significance values for the KEGG pathways for the UC dataset. Click here for file

Additional file 7 Comparison of the average and standard deviation for all pathways with 1,000 (black), 2,000 (blue) and 5,000 (green) permutation tests for row and column permutations separately (UC dataset). Click here for file

Additional file 8 Overview of the convergence of p-values with increasing permutation test number for the pathway “RNA polymerase” in the UC dataset. Click here for file

Additional file 9 All significant genes on the core networks for UC as well as DCM. Click here for file
