# Supplementary figures and images for: Systematic permutation testing in GWAS pathway analyses: identification of genetic networks in dilated cardiomyopathy and ulcerative colitis
Source: BMC Genomics. 2014 Jul 22;15:622. doi: 10.1186/1471-2164-15-622 (PMC4223581; doi:10.1186/1471-2164-15-622)

Figure S1

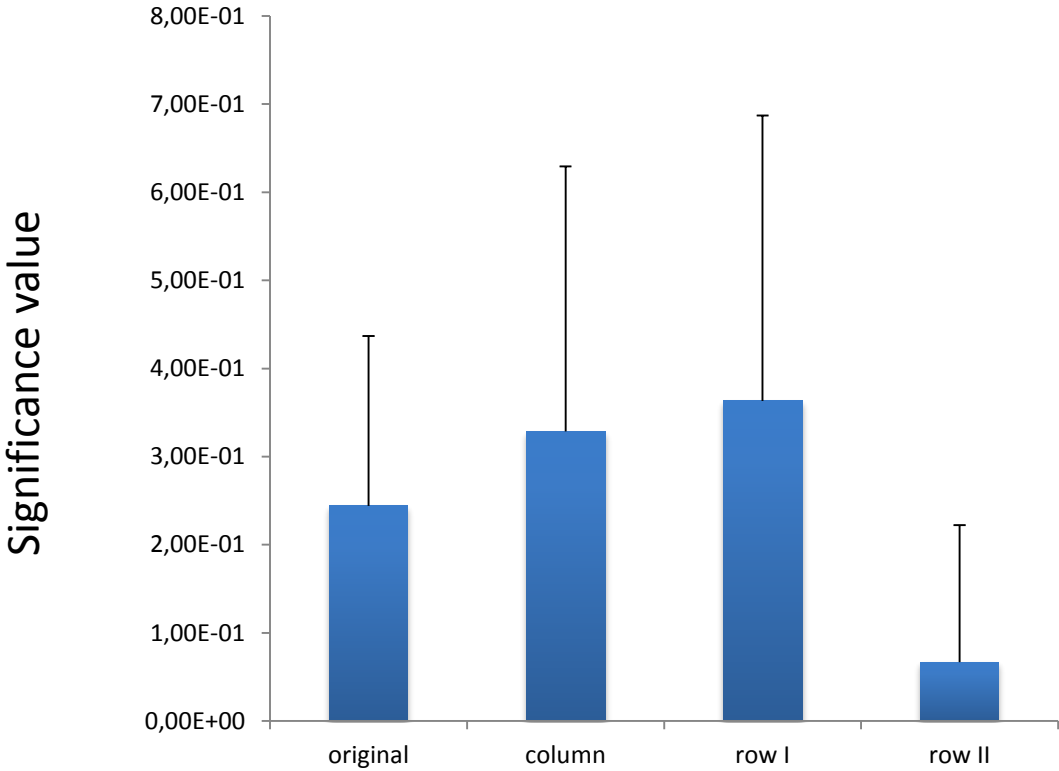

Supplement: Additional file 1 — Overview of the significance values resulting from the four sets of pathway analysis as bar chart. [file 1471-2164-15-622-S1.pdf]

Figure S2

Significant just in original p-values

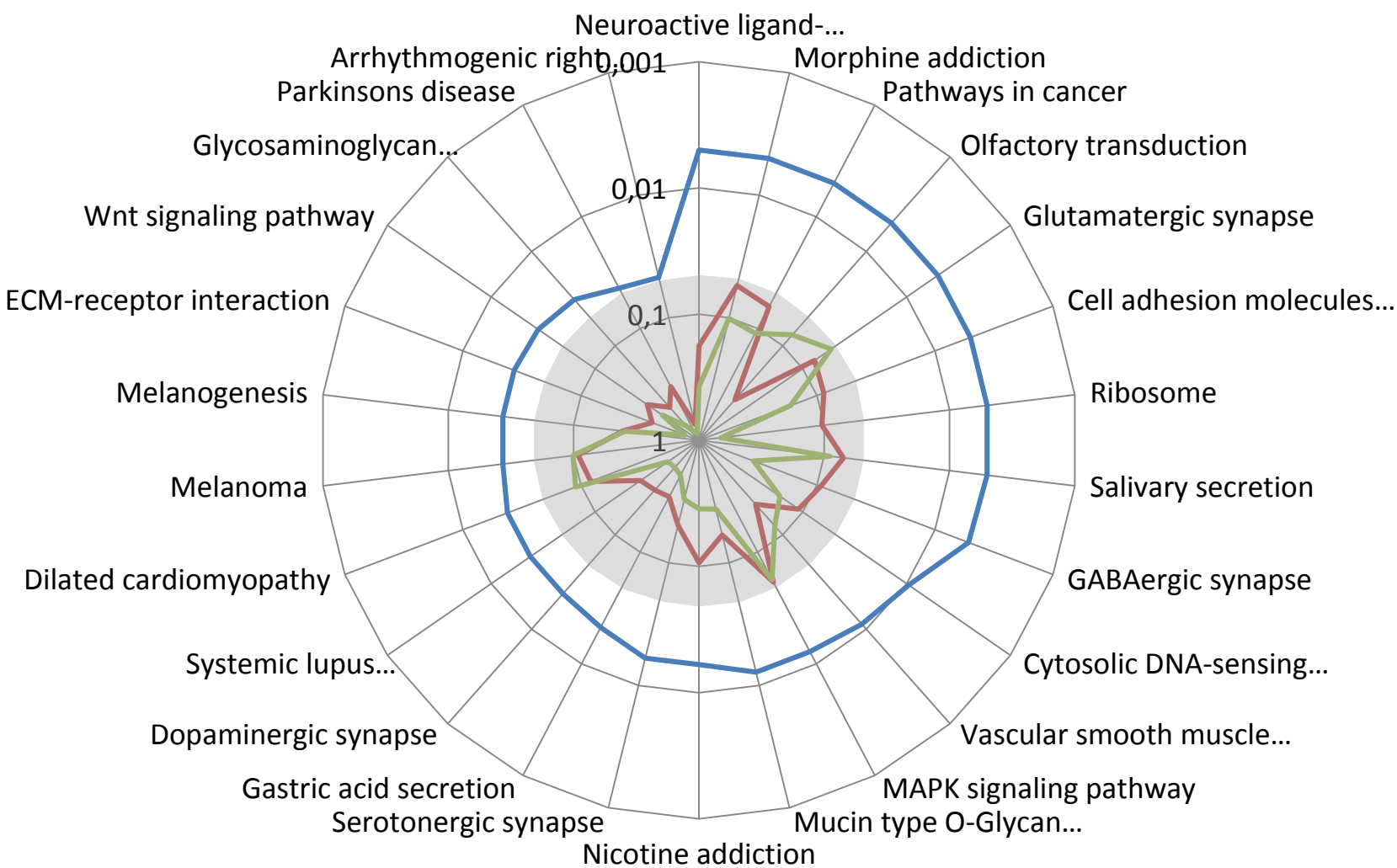

Supplement: Additional file 3 — Spider diagram of 26 pathways that have been excluded by both permutation approaches, being significant just in the original data set results. [file 1471-2164-15-622-S3.pdf]

Figure S3

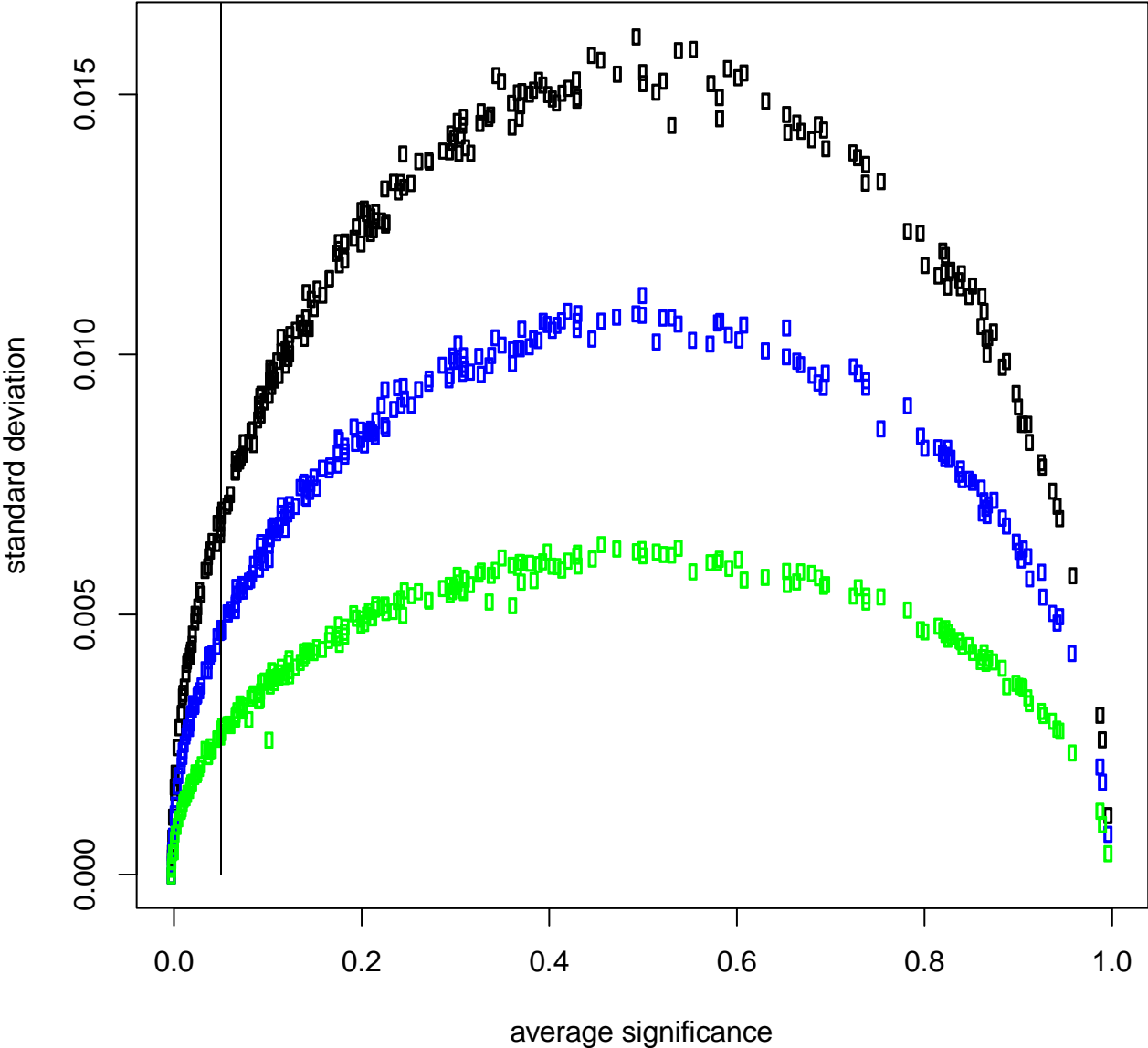

Supplement: Additional file 5 — Comparison of the average and standard deviation for all pathways with 1,000 (black), 2,000 (blue) and 5,000 (green) permutation tests for row and column permutations separately (DCM dataset). [file 1471-2164-15-622-S5.pdf]

Figure S4

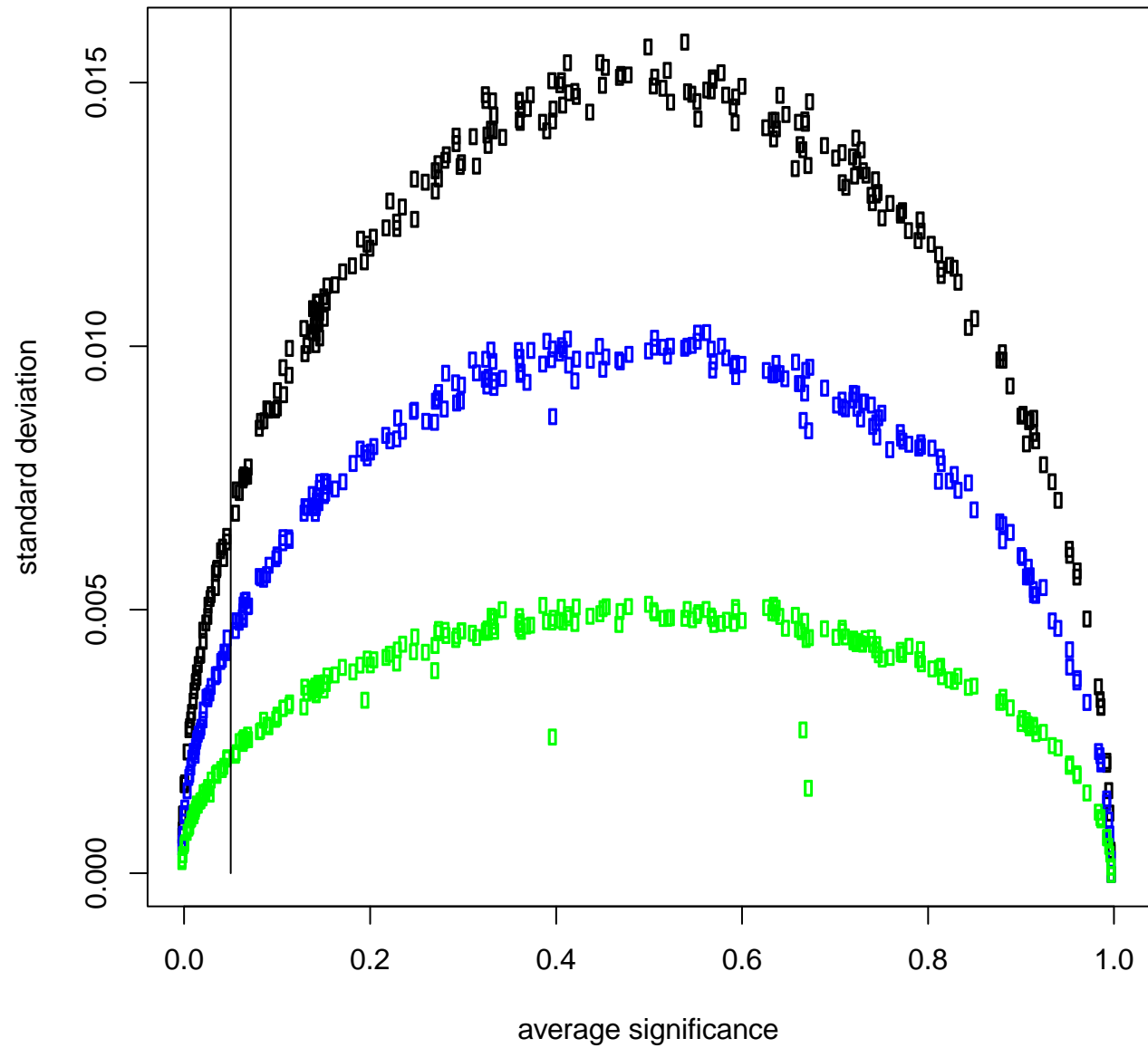

Supplement: Additional file 7 — Comparison of the average and standard deviation for all pathways with 1,000 (black), 2,000 (blue) and 5,000 (green) permutation tests for row and column permutations separately (UC dataset). [file 1471-2164-15-622-S7.pdf]

Figure S5

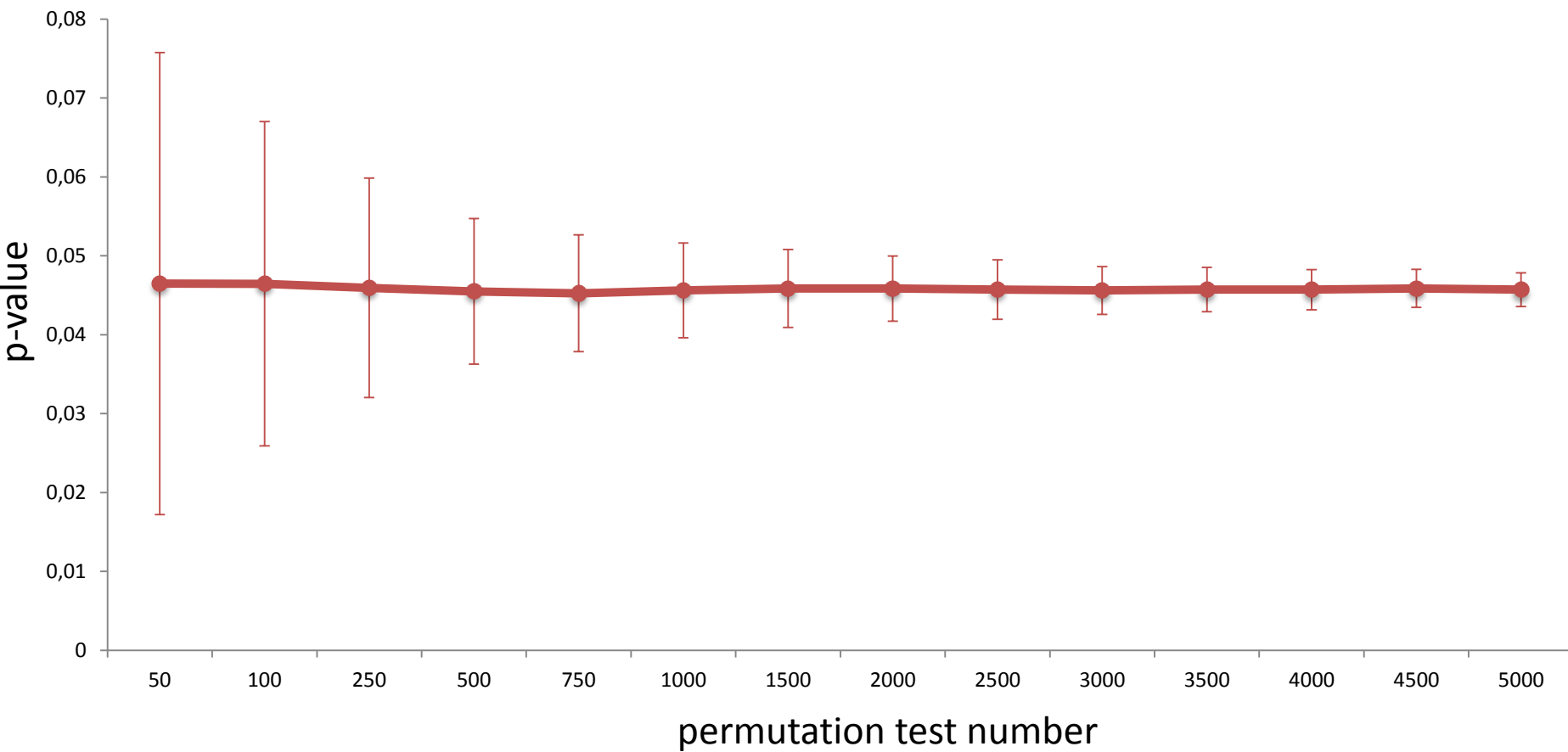

Supplement: Additional file 8 — Overview of the convergence of p-values with increasing permutation test number for the pathway “RNA polymerase” in the UC dataset. [file 1471-2164-15-622-S8.pdf]
